# 5-Aminolevulinic acid overcomes hypoxia-induced radiation resistance by enhancing mitochondrial reactive oxygen species production in prostate cancer cells

**DOI:** 10.1038/s41416-022-01789-4

**Published:** 2022-04-01

**Authors:** Takuya Owari, Nobumichi Tanaka, Yasushi Nakai, Makito Miyake, Satoshi Anai, Shingo Kishi, Shiori Mori, Rina Fujiwara-Tani, Yudai Hojo, Takuya Mori, Masaomi Kuwada, Tomomi Fujii, Masatoshi Hasegawa, Kiyohide Fujimoto, Hiroki Kuniyasu

**Affiliations:** 1 Department of Urology, Nara Medical University, Kashihara, Nara Japan; 2 Department of Molecular Pathology, Nara Medical University, Kashihara, Nara Japan; 3 Department of Pathology, Nara Medical University, Kashihara, Nara Japan; 4 Department of Radiation Oncology, Nara Medical University, Kashihara, Nara Japan

**Keywords:** Radiotherapy, Prostate cancer

## Abstract

**Background:**

The naturally occurring amino acid 5-aminolevulinic acid (5-ALA) is a precursor of protoporphyrin IX (PpIX) biosynthesised in the mitochondria. When accumulated PpIX is excited by light (wavelength of 625–635 nm), reactive oxygen species (ROS) are generated. Here, we investigated whether 5-ALA may increase the sensitisation of prostate cancer (PCA) cells to radiotherapy through the generation of ROS via its metabolite, PpIX.

**Methods:**

Effect of 5-ALA on PC-3 and DU-145 PCA cell lines treated with ionising radiation (IR) was examined in vitro and in vivo with assessment by clonogenic assay, mitochondrial function and ROS production under normoxia or hypoxia condition.

**Results:**

5-ALA enhanced intra-mitochondrial ROS production immediately after exposure to IR and decreased mitochondrial membrane potential via increase of intra-cellular PpIX. IR with 5-ALA induced mitochondrial dysfunction and increased ATP production, switching energy metabolism to the quiescence. Under hypoxic condition, ROS burst and mitochondrial dysfunction were induced by IR with 5-ALA resulting reducing cancer stemness and radiation resistance.

**Conclusion:**

These results suggest that combined therapy with 5-ALA and radiation therapy is a novel strategy to improve the anti-cancer effects of radiation therapy for PCA.

## Introduction

Prostate cancer (PCA) is the most common cancer in men. The estimated lifetime risk is 13%, in which the mortality is 20% [1, 2]. Radiation therapy (RT) is a curative primary treatment for patients with localised PCA [3, 4]; however, 5.3–12.6% of patients with high-risk PCA after primary RT and 50% of patients after salvage RT experienced biochemical recurrence [5–7].

Ionising radiation (IR) induces cancer death in two different mechanisms: a direct effect by DNA damage and an indirect anti-tumour effect by generating reactive oxygen species (ROS), which mainly comprise hydroxyl radicals, through water radiolysis reaction with oxygen. Radiation-induced ROS destabilise cancer cell integrity and DNA damage [8]. The levels of ROS and the adaptive antioxidant defense system are associated with resistance to RT for PCA [9].

Hypoxia has been defined as one of the most important causes of RT resistance via several mechanisms. Devascularisation reduces ROS production [10, 11]. Hypoxia-inducible factor-1 (HIF-1) reduces ROS production under by reprogramming of mitochondrial energy metabolism [12, 13]. Moreover, hypoxic conditions induce cancer stem cells (CSCs), which have been defined as critical drivers of tumour progression and metastasis [14, 15].

Several sensitisers of RT to overcome hypoxia-induced RT resistance by enhancing intra-mitochondrial ROS have been demonstrated [16–18]. The naturally occurring amino acid 5-aminolevulinic acid (5-ALA), a precursor of haem biosynthesis, is synthesised from succinyl coenzyme A and glycine. Exogenously administered 5-ALA can be enzymatically converted into protoporphyrin IX (PpIX) in mitochondria via the haem biosynthetic pathway. 5-ALA has been used as a photosensitiser in photodynamic therapy (ALA-PDT) [8]. The possibility that accumulated PpIX may enhance radiosensitivity has been pointed out for a long time, and it has been suggested that it may develop anti-tumour effects through activation of peripheral benzodiazepine receptors and generation of hydroxyl radicals [19, 20].

In the present study, we prompted to establish a novel strategy using 5-ALA to sensitise PCA cells to RT. For this, we performed targeting mitochondrial dysfunction by induction of mitochondrial ROS by ALA-PDT. We also investigated the mechanism of RT resistance induced by hypoxia focusing the metabolic response to hypoxia for establishing a method overcoming hypoxia-induced RT.

## Materials and methods

### Cells

PC-3 and DU-145 human PCA cell lines and MyC-CaP mouse PCA cell line were purchased from American Type Culture Center (Manassas, VA, USA). Cells were cultured in Dulbecco’s modified Eagle’s medium (DMEM), supplemented with 10% fetal bovine serum and 2% penicillin/streptomycin at 37 °C, 5% CO_2_ conditions.

### Cell treatment

Cells were pre-treated with 1 mM 5-ALA (a gift from SBI Pharmaceuticals Co., Ltd. (Tokyo, Japan) for 4 h, followed by treatment with X-ray IR using 150 kVp X-ray generator (Model MBR-1520R; Hitachi, Tokyo, Japan) as previously described [21].

### Reagents

Cobalt (II) chloride (CoCl_2_) and ammonium iron (II) sulfate hexahydrate (Fe^2+^)(FUJIFILM Wako Pure Chemical, Osaka, Japan), N-acetyl-L-cysteine (NAC, Sigma–Aldrich (St. Louis, MO) and deferoxamine (DFO, Cayman Chemical, Ann Arbor, MI, USA) were purchased.

### Accumulated intra-cellular PpIX quantification

The accumulated intra-cellular PpIX in 5-ALA-treated cells was quantified using a microplate spectrophotometer (Infinite 200 M PRO, Tecan, Männedorf, Switzerland) equipped with i-control (version 1.8) software, as described previously [22]. The fluorescence intensity at 635 nm was measured using an excitation wave (400 nm) as accumulated PpIX. PpIX fluorescence image was observed by BZ-X710 (KEYENCE, Osaka, Japan).

### Clonogenic assay

Colony survival was assessed using clonogenic assay to quantify cell survival after treatment according to our previous report [22]. Colonies containing more than 50 cells were counted as viable.

### Mitochondrial ROS and membrane potential measurement

To analyse mitochondrial ROS production and mitochondrial membrane potential (MMP) after treatment, MitoSOX (mitochondrial superoxide, Thermo Fisher Scientific, Waltham, MA, USA) and Si-DMA (mitochondrial singlet oxygen, DOJINDO, Kumamoto, Japan) were used. JC-1 assay (Cayman Chemical, Ann Arbor, MI, USA) was performed to determine MMP. The images fluorescence intensities were analysed by BZ-X710 (KEYENCE) at 1, 6 and 12 h after treatment.

### Apoptosis assay

Apoptotic cells were evaluated using MEBCYTO Apoptosis Kit (MBL, Nagoya, Japan) according to the annexin-based manufacturer’s protocol. Apoptotic cells were analysed immediately using a FACSCaliburTM flow cytometer (Becton-Dickinson, Franklin Lakes, NJ, USA).

### Seahorse assay

To analyse mitochondrial respiration and ATP production, an Extracellular Flux Analyzer XFp (Agilent Technologies, Santa Clara, CA, USA) was used. Assay was performed according to our previous report [23].

### Immunoblot analysis

Whole-cell protein was extracted from the cells 24 h after treatment using the assay buffer with protease inhibitor cocktail (Nacalai Tesque, Kyoto, Japan). The extracted proteins (20–40 µg) were subjected to immunoblot analysis according to our previous report [24]. Primary antibodies (1/1000) used for detecting were as follows: against B-cell lymphoma 2 (BCL-2)-associated X protein (BAX), BCL-2 (D55G8) and Glut1 (D3J3A)(Cell Signaling Technology, Danvers, MA, USA); BCL-2-associated agonist of cell death (BAD, C-2), BCL extra-large (BcL-xL, H-5) and β-actin (AC-15) (Santa Cruz Biotechnology, Dallas, CA, USA); HIF-1 alpha (EP1215Y) (Abcam, Cambridge, UK).

### Tumour-bearing syngeneic mouse model

This animal study was approved by the Committee on Animal Research of Nara Medical University (approval no. 12733, 2020/1/17). All animal experiments were conducted in accordance with the Guidelines for Welfare of Animals in Experimental Neoplasia. FVB/NJcl mice (5 week old, male, CLEA Japan Tokyo, Japan) were used. For a subcutaneous tumour model, syngeneic MyC-CaP cells (1 × 10^5^) in matrigel (BD Bioscience, San Jose, USA) were inoculated into the pelvic subcutaneous.

Treatments were initiated at day 14 when tumours reached 1000 mm^3^. Mice were divided into four groups (six mice each): normal control, 5-ALA alone, IR alone and IR with 5-ALA. Mice were administered 2 Gy/fraction (1 Gy/min) for 10 days under body protection using a lead collimator. In the IR with 5-ALA group, 5-ALA (30 mg/kg) was administered orally 3 h before irradiation in the dark phase to avoid PDT. Tumour volumes were calculated using the standard formula: 0.52 × (long diameter) × (short diameter) × 2. Mice were euthanised at 4 h (①) (*n* = 3) and day 7 (②) (*n* = 3) after treatment.

### Immunohistochemistry

Tumours excised at 4 h were fixed in 4% paraformaldehyde for 24 h and paraffin-embedded. Consecutive sections were processed for immunohistochemical analysis of primary antibodies (1/100) as follows: Ki-67 (SP6, Abcam), hydroxynonenal (4HNE, Abcam) and cleaved-caspase-3 (Cell Signaling). Positive cells were counted in five high-power fields of three sections from each group.

### Hypoxic conditions

To establish hypoxic conditions at varying oxygen tensions, we used BIONIX-1 hypoxic culture kit (Sugiyamagen, Tokyo, Japan). CoCl_2_ was used to mimic some hypoxic responses, including upregulation of HIF-1 [25].

### Reverse transcription-polymerase chain reaction (RT-PCR)

RT-PCR was performed according to our previous report [26]. The primer sets are listed in Supplementary Table 1. The thermocycler settings were 25 cycles of 96, 64 and 72 °C/30 s, followed by 72 °C/10 min.

### Sphere formation assay

To perform sphere formation assay, single cells collected immediately after different treatments were cultured according to our previous report [27].

### Detection of intra-mitochondrial Fe^2+^ under hypoxic conditions

To detect the accumulation of Fe^2+^ in mitochondria, Mito-FerroGreen (Doujindo, Kumamoto, Japan) was used according to the manufacturer’s protocol. Fluorescence images were captured using BZ-X710 (KEYENCE).

### Statistical analysis

Statistically significant differences were analysed using two-tailed Student’s *t*-test or one-way analysis of variance using GraphPad Prism 7.00 (GraphPad Software, San Diego, CA, USA). A *p*-value of <0.05 was considered to indicate statistical significance.

## Results

### Effect of 5-ALA on PpIX accumulation in PCA cells

Intra-cellular accumulation of PpIX in PCA cells was measured using a microplate spectrophotometer. The peak fluorescence intensity of PpIX with an excitation wavelength of 400 nm was observed at 635 nm. Intra-cellular accumulation of PpIX in PC-3 and DU-145 cells was observed after treatment with 5-ALA (1 mM, 4 h) (Fig. S1A). To determine whether PpIX localised in mitochondria, cells labeled with a mitochondrial dye, MitoGreen (PromoCell, Heidelberg, Germany), with or without 5-ALA treatment, were imaged by fluorescence microscopy. As shown in Fig. S1B, 5-ALA led to the biosynthesis of PpIX in mitochondria of PCA cells.

### Effect of 5-ALA on the sensitivity of PCA cells to RT

**Fig. 1:**
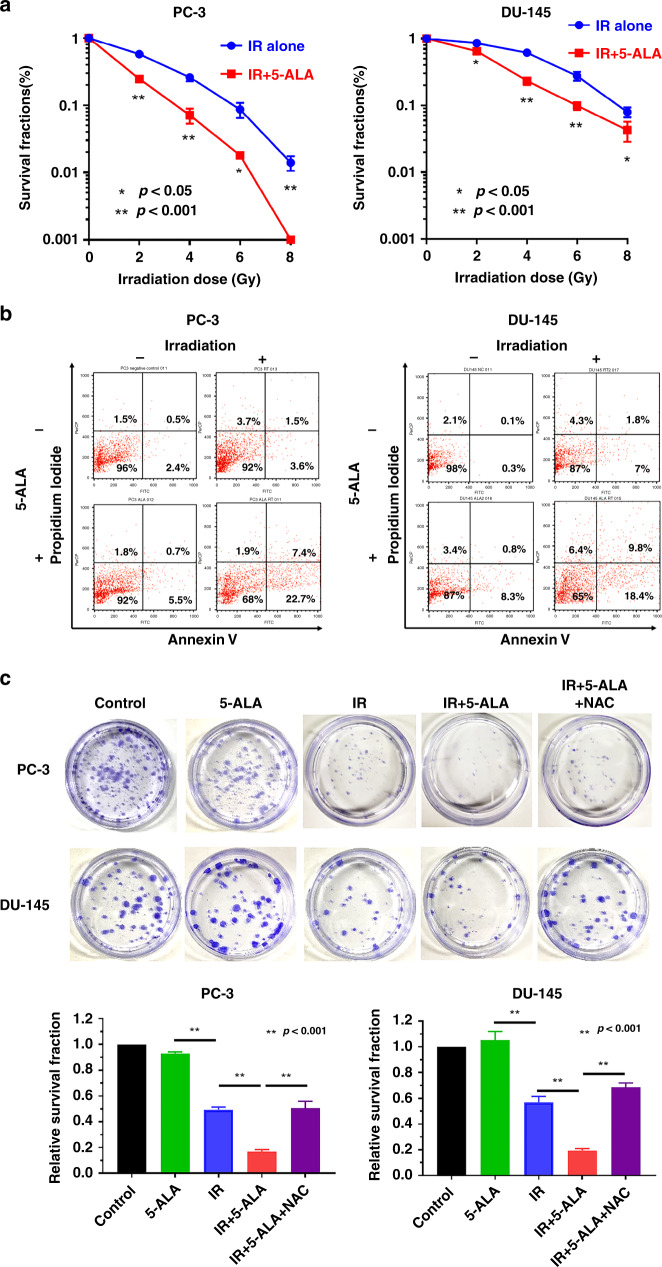
Effect of 5-ALA on the sensitivity of PCA cells to RT. **a** Effect of pre-treatment with 5-ALA (1 mM) for 4 h on sensitivity to ionising radiation (IR) was examined by clonogenic assay in PC-3 and DU-145 cells. **b** Cell apoptosis analysed by flow cytometry after different treatments. **c** Colony survival after different treatments including pre-treatment with ROS inhibitor, NAC (500 μM) for 24 h. Dose of IR: 3 Gy. Statistical analysis; by Student’s *t*-test (mean ± SEM, *n* = 3).

To evaluate the effect of 5-ALA as a radiosensitiser of castration-resistant PCA cells, survival of cells after different treatments was evaluated using clonogenic assay. As shown in Fig. 1a, treatment with 5-ALA led to a reduction in colony survival after exposure to IR compared with IR alone in PC-3 and DU-145 cells. Furthermore, the apoptosis-inducing effect of 5-ALA was evaluated using annexin V-FITC and PI staining by flow cytometry, and 5-ALA was found to increase the apoptotic rates 24 h after exposure to IR compared with IR alone (5-ALA + IR vs. IR alone, 30.1 ± 1.2% vs. 5.1 ± 0.2% and 28.2 ± 1.5% vs. 8.8 ± 0.2% in PC-3 and DU-145 cells, respectively) (Fig. 1b). Most importantly, as shown in Fig. 1c, the reductions in survival of PC-3 and DU-145 cells treated with 5-ALA was recovered by pre-treatment with the ROS inhibitor NAC (500 μM), suggesting that the main mechanism of 5-ALA-mediated radiosensitisation may be attributed to ROS production.

### Effect of 5-ALA on intra-cellular mitochondrial ROS production and MMP

**Fig. 2:**
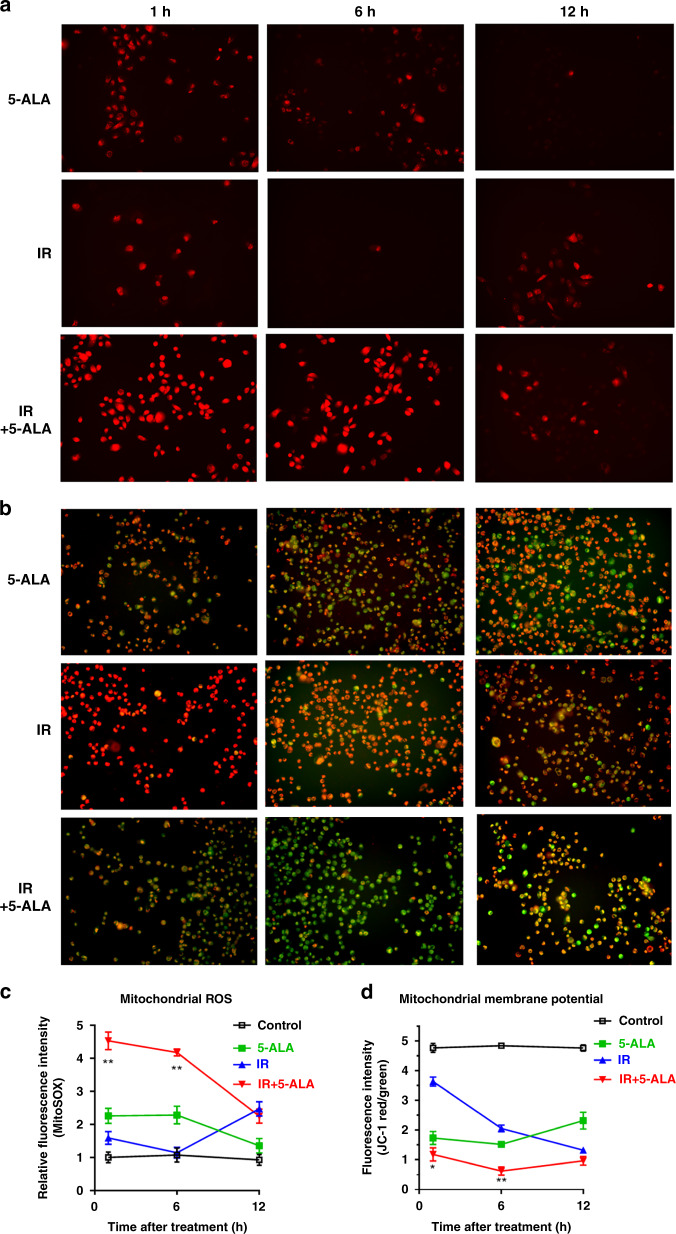
Effect of 5-ALA on intra-cellular mitochondrial ROS production and MMP after RT in PC-3 cells. **a** ROS production in mitochondria after different treatments performed using MitoSOX. **b** Mitochondrial membrane potential performed by JC-1 staining. Merging of J-aggregates (red) and J-monomers (green) has been shown. Apoptotic cells and depolarised mitochondria are detected by the green color. **a**, **b** Images were taken at 1, 6, and 12 h after radiation. **c** Relative fluorescence intensity of MitoSOX. Statistical analysis; by Student’s *t*-test (mean ± SEM, *n* = 5). **d** Relative fluorescence intensity of JC-1 staining (red color/green color intensity). Statistical analysis; by Student’s *t*-test (mean ± SEM, *n* = 5). *p < 0.05, **p < 0.001. IR ionising radiation.

MitoSOX^TM^ Red mitochondrial superoxide indicator was used and JC-1 staining was performed for real-time monitoring of intra-mitochondrial ROS production and MMP. The peak fluorescence intensity of mitochondrial ROS was observed 12 h after exposure to IR alone. In contrast, IR with 5-ALA treatment exhibited intra-mitochondrial ROS burst immediately after exposure to IR, and the peak fluorescence intensity continued to 6 h after treatment. The level of mitochondrial ROS in the IR with 5-ALA group during 1–6 h after treatment was significantly higher among all groups in PC-3 (Fig. 2a, c) and DU-145 (Fig. S2A, C) cells. It has been demonstrated that the loss of MMP occurs during the induction of apoptosis [28]. The green monomer of JC-1 could enter the cytoplasm and aggregate in normal mitochondria, with the formation of numerous red J-aggregates, and the fluorescence transition from red to green suggested the loss of MMP and thus significant mitochondrial damage. Representative merged photographs of J-aggregates (green) and J-monomers (red) are shown in Fig. 2b and Fig. S2B. IR with 5-ALA treatment significantly decreased MMP (red/green fluorescence intensity) among all groups along with increased intra-mitochondrial ROS production during 1–6 h after treatment in PC-3 (Fig. 2b, d) and DU-145 (Fig. S2B, D) cells, suggesting that mitochondrial ROS burst induced by IR with 5-ALA treatment caused a reduction in MMP, which promoted apoptosis.

### Effect of combined treatment of RT with 5-ALA on mitochondrial function

**Fig. 3:**
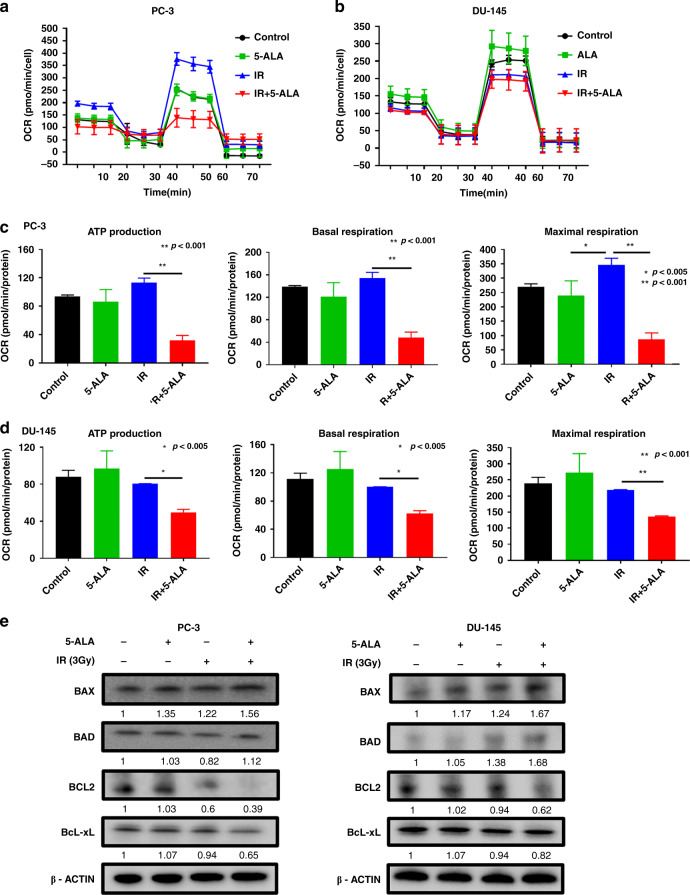
Effect of combined treatment of RT with 5-ALA on mitochondrial function and expression of apoptosis-related proteins in PCA cells. **a**, **b** Mitochondrial oxygen consumption rate (OCR). **c**, **d** Mitochondrial ATP production, basal respiration and maximal respiration after different treatments. Student’s *t*-test (mean ± SEM, *n* = 3). **e** Western blotting for the expression of BCL-2 family proteins, mitochondrial apoptosis-related proteins, including pro-apoptotic proteins (BAX and BAD) and anti-apoptotic proteins (BCL-2 and BcL-xL) 24 h after treatments. IR ionising radiation.

Mitochondrial dysfunction plays a key role in the induction of apoptosis. Several studies have demonstrated that increased mitochondrial ROS exhibits a suppressive effect on mitochondrial function, eventually inducing apoptosis and developing sensitivity to RT in cancer cells [16, 17]. To evaluate the effect of IR with 5-ALA treatment on mitochondrial metabolic function, we investigated the mitochondrial OCR value in different respiratory states after each treatment using Extracellular Flux Analyzer XFp. OCR of PC-3 and DU-145 cells in the IR alone group exhibited opposite effects (Fig. 3a, b); OCR of PC-3 cells was increased by IR alone, but OCR of DU-145 cells was decreased. This result might be affected by the nature of the PCA cell lines. Jayakumar S et al. demonstrated that the level of ROS inducible by IR in DU-145 cells was lower than that in PC-3 cells, and it was one of the major mechanisms of RT resistance in DU-145 cells [29]. In contrast, OCR of the IR with 5-ALA group was significantly decreased among all groups in both PC-3 and DU-145 cells. In particular, this effect was remarkable in PC-3 cells (Fig. 3a, b). IR with 5-ALA treatment led to a significant reduction in the basal and maximal OCR and ATP production in PC-3 and DU-145 cells among all groups (Fig. 3c, d). These results indicated that intra-mitochondrial ROS burst induced by IR with 5-ALA caused a reduction in ATP production and shifted the mitochondrial energy metabolism from oxidative phosphorylation (OXPHOS) status to the quiescent status.

### Effect of combined treatment of RT with 5-ALA on expression of apoptosis-related proteins

Next, the expression of mitochondria-mediated apoptotic signal proteins was evaluated using immunoblotting. BCL-2 family protein regulates the outer MMP and mitochondria-mediated apoptosis. As shown in Fig. 3e, the expression level of the pro-apoptotic BAX and BAD increased 24 h after treatment with IR and 5-ALA. Conversely, the expressions of anti-apoptotic BCL-2 and BcL-xL were significantly inhibited.

### Effect of combined treatment of RT with 5-ALA in subcutaneous tumour-bearing syngeneic mouse model

**Fig. 4:**
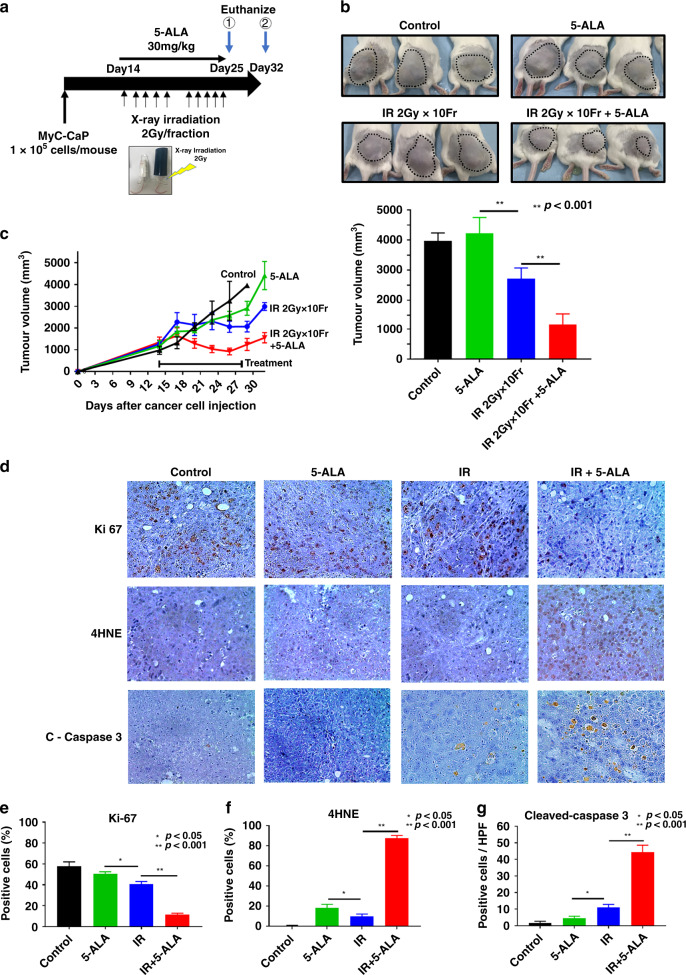
In vivo anti-cancer effects of the combined therapy with 5-ALA and radiation. **a** The animal study protocol. MyC-CaP cells (1 × 10^5^) are inoculated in the pelvic subcutaneous tissue. Radiation dose (2 Gy/fraction, 2 times/day for 5 consecutive days), Further, 5-ALA (30 mg/kg) is administered orally 3 h before ionising radiation (IR). **b** Photographs of mice at euthanasia (②) and at 7 days after IR treatment. **c** Alteration of tumour volume (mean ± SEM, *n* = 3). Right panel—Tumour volume at euthanasia. **d–g** Immunohistochemistry for Ki-67, 4HNE and cleaved-caspase-3 in tumours at the time of euthanasia (①) and at 4 h. Positive cells in five randomised fields (mean ± SEM, *n* = 3 mice each) have been shown.

The treatment protocol to evaluate the therapeutic effect in vivo is shown in Fig. 4a. A MyC-CaP tumour-bearing syngeneic mouse model was used to evaluate the anti-tumour effect of IR with 5-ALA compared with IR alone in vivo. MyC-CaP cells (1 × 10^5^ cells per mouse) were injected into the subcutaneous tissue at the pelvic area of the male FVB/NJcl mice. Mice were randomly divided into four different groups: normal control, 5-ALA alone, IR alone and IR with 5-ALA. The 5-ALA alone group showed no anti-tumour effect (Fig. 4b, c). IR prevented tumour growth, and the reduction rate was ~50% compared with the normal control and 5-ALA alone groups. Moreover, the therapeutic effect of IR was further enhanced by the addition of orally administered 5-ALA. However, it did not eliminate the tumour. Further, 4 h after treatment, three of the six mice were euthanised (①) and tumour tissues were excised (Fig. 4d, e). Immunohistochemical analysis of Ki-67, 4HNE and cleaved-caspase-3 was performed to investigate tumour proliferation, ROS production and apoptosis in the different groups. The number of Ki-67-positive cells was significantly decreased in the IR with 5-ALA group. To evaluate ROS production in vivo, 4HNE immunohistochemical analysis, a commonly used marker for oxidative stress, was used. The expression of 4HNE was dramatically increased in the IR with 5-ALA group. Moreover, cleaved-caspase-3, an apoptosis marker that plays a key role in apoptotic cell death, was activated by treatment with IR and 5-ALA compared with IR alone. These results suggested that increased ROS production after IR was enhanced by the addition of orally administered 5-ALA to cause apoptotic cell death in vivo.

### Effect of combined treatment of RT with 5-ALA on hypoxia-induced radiation resistance

**Fig. 5:**
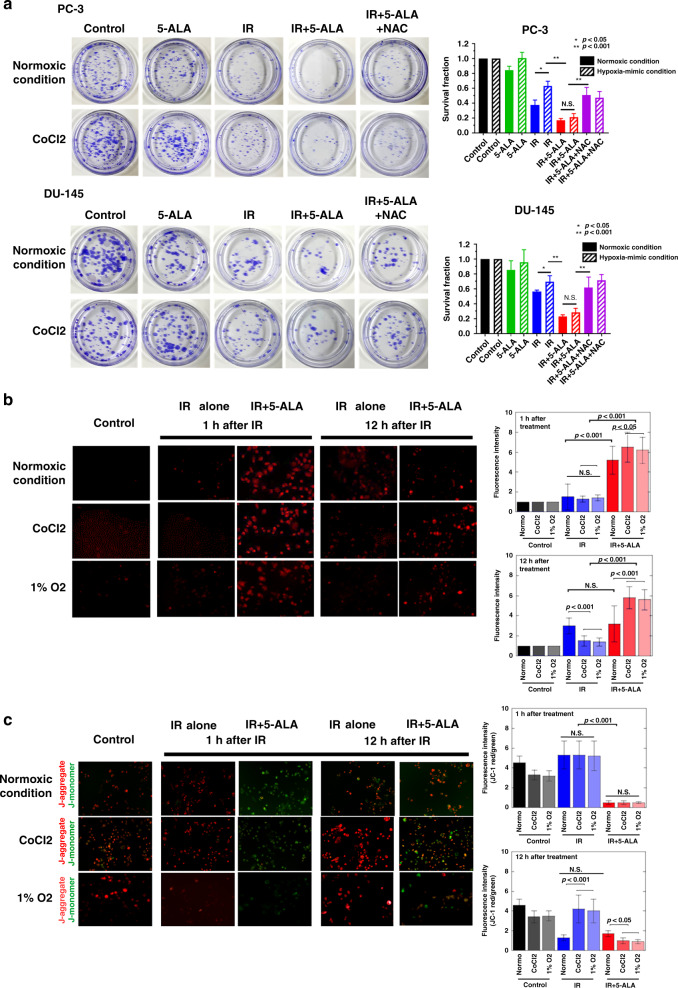
Effects of 5-aminolevulinic acid (5-ALA) as a radiosensitiser under hypoxic conditions in PCA cells. **a** Anti-tumour effects of different treatments under hypoxia investigated by clonogenic assay. Treatment with CoCl_2_ (100 μM) at pre- and post-RT for 24 h acts as a hypoxia-mimicking condition. **b** Intra-mitochondrial ROS production analysed by MitoSOX 1 and 12 h after radiation under hypoxia by 1% O2 or CoCl_2_ (100 μM). Relative fluorescence intensity of MitoSOX. Statistical analysis; by Student’s *t*-test (mean ± SEM, *n* = 5). **c** Mitochondrial membrane potential evaluated by JC-1 staining at 1 and 12 h after radiation under hypoxia by 1% O_2_ or CoCl_2_ (100 μM). Relative fluorescence intensity of JC-1. Statistical analysis; by Student’s *t*-test (mean ± SEM, *n* = 5). IR ionising radiation.

Hypoxia is a common feature of solid tumours [30] and is one of the most important causes of radiation resistance in PCA cells [31]. We investigated whether 5-ALA exerted an additional therapeutic effect on RT for PCA cells under hypoxic conditions. We produced hypoxia-mimicking conditions using CoCl_2_ at a concentration of 100 μM before and after treatment for 24 h to avoid reoxygenation during treatment. Clonogenic assay demonstrated that the reduction in cell survival in the IR alone group was significantly lower under CoCl_2_-treated conditions than under normoxic conditions. Conversely, similar to normoxic conditions, 5-ALA enhanced radiosensitivity under CoCl_2_-treated conditions in PC-3 and DU-145 cells (Fig. 5a). Moreover, as expected, we demonstrated that enhanced radiosensitivity by 5-ALA was inhibited by pre-treatment with the ROS inhibitor NAC (500 μM). These results indicated that 5-ALA exerted a therapeutic effect of enhancing radiosensitivity under hypoxic conditions by enhancing ROS production.

Next, we investigated whether IR with 5-ALA could induce mitochondrial damage caused by intra-mitochondrial ROS burst under hypoxic conditions, similar to the results of normoxic conditions. An intrinsic feature of hypoxia is the lack of oxygen, resulting in less ROS production by IR [11, 18]. First, we investigated mitochondrial ROS production after RT (Fig. 5b). As shown previously, with respect to IR alone, the peak fluorescence intensity of mitochondrial ROS was observed at 12 h after exposure to IR. The peak fluorescence intensity after IR was significantly lower under the hypoxia conditions (CoCl_2_ and 1% O_2_) than under normoxic conditions. Conversely, it was interesting to observe that 5-ALA could sustain higher mitochondrial ROS production at 12 h after exposure to IR under hypoxia conditions (CoCl_2_ and 1% O_2_) than under normoxic conditions. Next, we evaluated the production of singlet oxygen, which is the main ROS produced by ALA-PDT, in mitochondria. Although mitochondrial singlet oxygen production was observed 1 h after exposure to IR with 5-ALA, it could not be detected at 12 h (Fig. S3A, B). Therefore, higher levels of mitochondrial ROS induced by IR with 5-ALA under hypoxic conditions comprised mitochondrial superoxide. Next, we compared MMP after IR between normoxic and hypoxia conditions (CoCl_2_ and 1% O_2_) using the MitoProbe ^TM^ JC-1 assay (Fig. 5c). Reflecting the reduction of mitochondrial ROS production, MMP after IR alone was significantly higher under hypoxia conditions (CoCl_2_ and 1% O_2_) than under normoxic conditions. Conversely, 5-ALA retained lower MMP levels under hypoxia conditions (CoCl_2_ and 1% O_2_) 12 h after exposure to IR.

### Effect of combined treatment of RT with 5-ALA on hypoxia-induced glycolysis

**Fig. 6:**
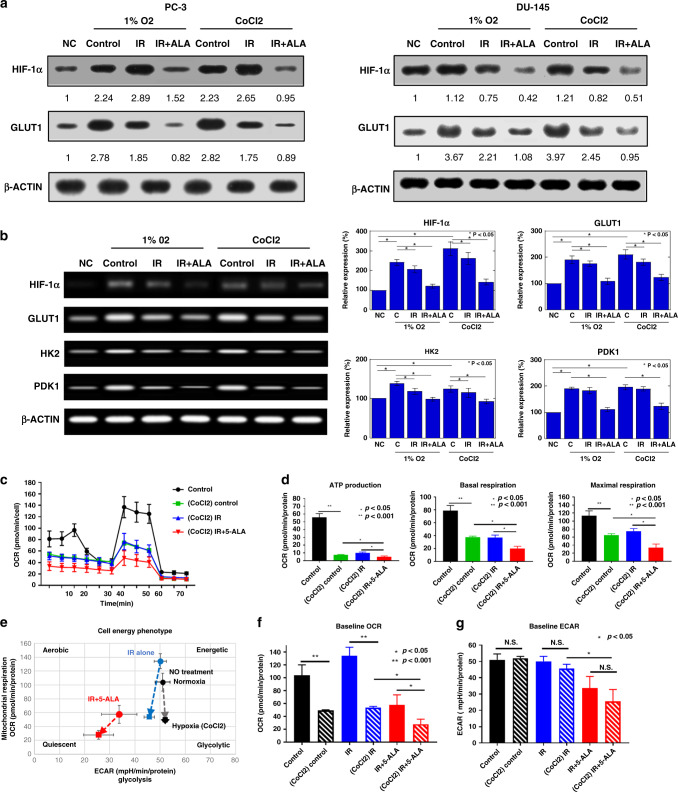
Effect of the combined therapy with 5-aminolevulinic acid (5-ALA) and radiation on mitochondrial energy metabolism in PCA cells. **a** Protein levels of HIF-1α and GLUT1 24 h after after radiation (IR) or IR + 5-ALA under hypoxia by 1% O_2_ or CoCl_2_ (100 μM). analysed by western blotting. **b** mRNA expressions of HIF-1α and glycolytic genes (GLUT1, HK2 and PDK1) in PC-3 cells measured by RT-PCR. **c** Mitochondrial oxygen consumption rate (OCR) of PC-3 cells after treatments. **d** Mitochondrial ATP production, basal respiration and maximal respiration of PC-3 after treatments in hypoxia (CoCl_2_). Statistical analysis; by Student’s *t*-test (mean ± SEM, *n* = 3). **e** Change in energy metabolism after treatments under normoxic (●) to hypoxic (■) conditions. **f** Baseline OCR and **g** extracellular acidification rate (ECAR) after treatments. IR ionising radiation.

The concept of tumour metabolism is characterised by the Warburg effect, which is attributed to the unique metabolism of cancer cells that shifts the ATP production from OXPHOS to glycolysis, even under normoxic as well as hypoxic conditions [32]. Under hypoxic conditions, HIF-1-mediated metabolic reprograming, especially the increased expression of glycolytic enzymes, including glucose transporter 1 (GLUT1), hexokinase 2 (HK2) and pyruvate dehydrogenase kinase 1 (PDK1), has been associated with antioxidant capacity and RT resistance [12, 33, 34]. To evaluate mitochondria-mediated metabolism after treatment under hypoxic conditions, we first investigated the expressions of HIF-1α, GLUT1, HK2 and PDK1. Western blotting demonstrated that the expressions of HIF-1α and GLUT1 were higher under hypoxic (1% O_2_ and CoCl_2_ treatment) conditions than under normoxic conditions (Fig. 6a). These upregulated protein levels were markedly decreased by IR treatment with 5-ALA. Similar to the results of western blotting, RT-PCR demonstrated that the upregulated expressions of mRNAs of HIF-1α and glycolytic enzymes, including GLUT1, HK2 and PDK1, were significantly inhibited by IR treatment with 5-ALA under hypoxic conditions (Fig. 6b). Next, to evaluate mitochondrial energy metabolism under hypoxia and the mechanism of the downregulated expression of glycolytic enzymes after treatment with IR and 5-ALA, we investigated mitochondrial ATP production, OCR and extracellular acidification rate (ECAR), which represents glycolysis, using Extracellular Flux Analyzer XFp. As shown in Fig. 6c, hypoxic conditions led to the reduction of OCR and ATP production. Moreover, IR with 5-ALA led to a dramatic reduction in OCR and ATP production (Fig. 6d). The changes in mitochondrial energy metabolism phenotype after different treatments from normoxic to CoCl_2_-treated conditions are shown in Fig. 6e. Although OCR under CoCl_2_-treated conditions was significantly lower than that under normoxic conditions, there were no significant differences in ECAR of normal control and IR alone groups between CoCl_2_-treated and normoxic conditions (Fig. 6f, g). These findings indicated that hypoxic conditions switched the mitochondria-mediated tumour metabolism to glycolysis due to the Warburg effect. In contrast, IR with 5-ALA led to the downregulation of both OCR and ECAR (Fig. 6f, g), demonstrating that mitochondrial metabolism was switched to the quiescent stage by treatment with IR and 5-ALA.

### Effect of combined treatment of RT with 5-ALA on hypoxia-induced cancer stemness

**Fig. 7:**
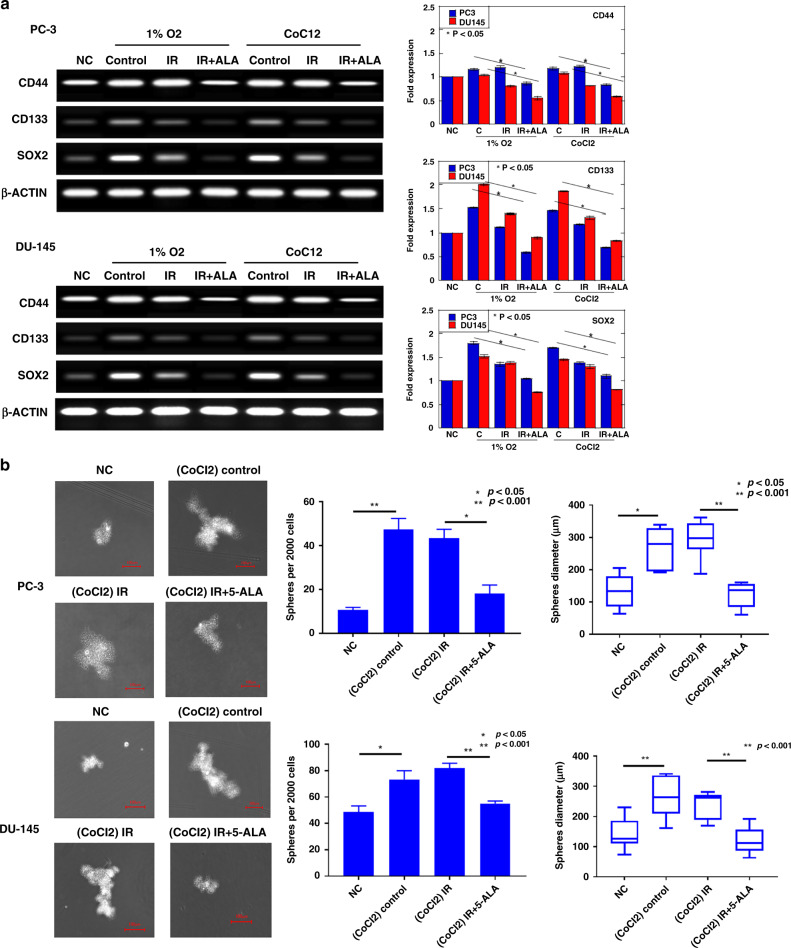
Effect of combined treatment of RT with 5-ALA on hypoxia-induced cancer stemness in PCA cells. **a** RT-PCR results showing the mRNA expression of stem cell markers including CD44, CD133 and SOX2 after radiation (IR) or IR + 5-ALA under hypoxia by 1% O_2_ or CoCl_2_ (100 μM). Statistical analysis; by Student’s *t*-test (mean ± SEM, *n* = 3). **b** Sphere formation after treatments under hypoxia. Statistical analysis; by Student’s *t*-test. IR ionising radiation.

Hypoxia and hypoxia-mediated glycolysis enhance CSC reprograming [35]. CSCs are regarded as major contributors to RT resistance [36]. We examined whether hypoxic conditions caused cancer stemness in PCA cells and whether IR and 5-ALA treatment could inhibit hypoxia-induced cancer stemness through the inhibition of hypoxia-induced glycolysis. We first measured the gene expression of cancer stemness-related factors, including CD44, CD133 and SOX2, which are major stemness markers of PCA [37, 38]. As shown in the RT-PCR data in Fig. 7a, the expression levels of CD44, CD133 and SOX2 under hypoxic conditions were significantly higher than those under normoxic conditions in PC-3 cells. Further, the expression levels of CD133 and SOX2 under hypoxic conditions were significantly higher than those under normoxic conditions in DU-145 cells. Most importantly, the upregulation of cancer stemness markers induced under hypoxic conditions was inhibited by IR with 5-ALA treatment. Subsequently, we performed a sphere formation assay and observed a significant reduction in sphere number and diameter in both PC-3 and DU-145 cells treated with IR and 5-ALA (Fig. 7b). Collectively, these findings suggest that IR with 5-ALA has the potential to inhibit hypoxia-induced cancer stemness through the inhibition of glycolysis.

### Effect of accumulated Fe^2+^ under hypoxia on efficiency of combined treatment of RT with 5-ALA

We demonstrated that exposure to hypoxic conditions (1% O_2_ or CoCl_2_ treatment) for 24 h induced the accumulation of Fe^2+^ in mitochondria (Fig. S4A). We hypothesise that the mechanism of the remain higher level of intra-mitochondrial ROS induced by IR with 5-ALA under hypoxic condition was associated with the intra-mitochondrial Fe^2+^ accumulation induced by hypoxia. To determine the correlation between intra-mitochondrial Fe^2+^ and the higher levels of ROS induced by IR with 5-ALA, we evaluated mitochondrial ROS production, MMP, and the therapeutic effect in normal culture medium and under Fe^2+^ (100 μM), CoCl_2_ and CoCl_2_ with iron chelator (DFO, 100 μM) conditions. First, we evaluated intra-mitochondrial ROS production 12 h after exposure to IR (Fig. S4B). Although fluorescence intensity after IR alone was significantly lower under CoCl_2_ than under normal conditions, there were no significant differences in fluorescence intensity between the normal and Fe^2+^ conditions. In contrast, fluorescence intensity after IR with 5-ALA treatment was significantly higher under Fe^2+^ and CoCl_2_ conditions than under normal conditions. Moreover, higher levels of ROS production under CoCl_2_ conditions were inhibited by DFO. Next, we evaluated MMP at 12 h after IR treatment under different conditions (Fig. S4C). IR with 5-ALA significantly decreased MMP simultaneously with increased intra-mitochondrial ROS production under Fe^2+^ and CoCl_2_ conditions than under normal conditions. The reduction of MMP under CoCl_2_ conditions was also inhibited by DFO. Moreover, as shown in Fig. S5, the additional therapeutic effect of 5-ALA under CoCl_2_ conditions was inhibited by DFO. These results indicated that Fe^2+^ could enhance the additional therapeutic effect of 5-ALA in RT for PCA through the activation of intra-mitochondrial ROS production. Thus, under hypoxic conditions, increased advantages of RT combined with 5-ALA could be obtained by Fe^2+^ accumulation in mitochondria.

## Discussion

RT is one of the primary treatment approaches for PCA. In the present study, our therapeutic strategy focused on enhancing mitochondrial ROS production, which plays a key role in the therapeutic efficacy of RT. We demonstrated that 5-ALA could be a radiosensitiser in PCA cells through increased intra-mitochondrial ROS production. ROS burst in mitochondria induced the loss of MMP and ATP production, resulting in mitochondria-mediated apoptosis.

5-ALA is widely applied to PDT by ROS generation via PpIX in mitochondria [8]. ALA-PDT has been approved for the treatment of superficial malignancy in skin, oesophageal and lung cancer [39]. However, the application of ALA-PDT for PCA is anatomically difficult [40]. In the present study, we evaluated the therapeutic potential of 5-ALA as a radiosensitiser for PCA. We demonstrated the accumulation of PpIX in PCA cells by 5-ALA treatment (Supplementary Fig. 1). We already demonstrated the accumulation of PpIX in urine sediments of PCA patients treated with 5-ALA [41]. In view of safety of patients, it is important that the excessive accumulation of PpIX in mitochondria is tumour-specific because ferrochelatase is inactive in tumour cells owing to the lack of electron supply from the tricarboxylic acid cycle due to the Warburg effect. Thus, normal tissues are protected from ALA damage [21].

In this study, it was shown that ALA-PDT mainly produced singlet oxygen [8, 42], whereas ALA-IR produced and maintained superoxide. In PDT, after 5-ALA administration PpIX accumulated in mitochondria is excited with a peak wavelength of 630–635 nm [43]. In contrast, the overproduction of ROS by X-ray IR at different wavelengths may be different from the mechanism of ALA-PDT. The generation of singlet oxygen by ALA-PDT induces necroptosis via RIP-3 [44]. In contrast, our observations showed that the effect of ALA-IR is increased in hypoxia, which is accompanied by an increase in intra-cellular Fe^2+^. Furthermore, the cytotoxic effect of IR was enhanced by Fe^2+^ loading, which was further enhanced by the combination of 5-ALA with IR. IR-activated Fe^2+^ induces Fenton reaction, which leads to superoxide formation and induces ferroptosis [45, 46]. It has been reported that 5-ALA induces ferroptosis in oesophageal cancer [47]. These findings suggest that 5-ALA-induced intra-cellular PpIX accumulation may promote IR-induced Fe^2+^-mediated superoxide production, resulting in ferroptosis. The mechanism of cytotoxicity induced by 5-ALA + IR is different from that induced by 5-ALA-PDT and needs to be investigated in more detail.

Mitochondria are the major site for ROS production and have been well-recognised as a treatment target for apoptosis [16, 48]. Although ROS production induced by IR mainly includes production of hydroxyl radicals, IR increased intra-mitochondrial ROS level through the upregulation of mitochondria electron transport chain and mitochondrial volume [49]. Our data showed that a peak level of intra-mitochondrial ROS was observed at 12 h after IR in the IR alone group. However, in the IR with 5-ALA group, dramatic increase in intra-mitochondrial ROS production was observed immediately after IR. Moreover, 5-ALA significantly decreased MMP and ATP production after IR. The decreased MMP and ATP synthesis led to further ROS production [50]. Moreover, intra-mitochondrial ROS burst and the loss of MMP triggered BAX translocation to mitochondria and downregulated BCL-2/BcL-xL. BAX decreases the permeability of the outer membrane, resulting in sensitivity to voltage-dependent anion channel deficiency and release of cytochrome C [50, 51]. Our findings suggest that IR with 5-ALA treatment caused the decrease of MMP and ATP production by ROS burst in mitochondria, upregulating BAX and downregulating BCL-2/BcL-xL, resulting in mitochondria-dependent apoptosis.

Hypoxia is characteristic in many solid tumours and the important risk factor of disease progression and recurrence after RT. In patients with PCA treated with RT, hypoxia is an independent predictor of biochemical and local recurrence [52]. Under hypoxic conditions, HIF-1 accelerates glycolysis and biogenesis of antioxidant agents [34]. Activation of HIF-1 and glycolysis induces CSCs, which contribute to RT resistance [35, 36, 52]. In the present study, hypoxia increased the expression of HIF-1 and glycolytic enzymes, resulting in the switch of energy metabolism from OXPHOS to glycolysis. Furthermore, hypoxic treatment promoted the stemness of PCA cells. CD44, CD133 and SOX2 are considered to be the major cancer stem cell markers in prostate cancer [38]. It has been shown that cancer stem cells in prostate cancer are strongly involved in RT resistance [53, 54]. Therefore, targeting of cancer stem cells is important to enhance the efficacy of radiotherapy over targeting of non-stem cells. In the present study, the combination of IR and 5-ALA reduced the expression of stem cell markers induced by hypoxia and suppressed sphere formation. These results suggest that the combination of IR and 5-ALA may help overcome hypoxia-induced RT resistance in prostate cancer.

Hypoxia and the HIF-1 activation led to enhanced Fe^2+^ accumulation in tumours [55]. We demonstrated that higher levels of Fe^2+^ accumulation in mitochondria was induced by hypoxic conditions (Fig. S4A). Fe^2+^ enhances the ROS production, and mitochondrial depolarisation, and then therapeutic effect of mitochondria-mediated PDT via Fenton reaction [56, 57]. Our data demonstrated that increase of intra-mitochondrial ROS production in hypoxia was abrogated by Fe chelator, DFO. Moreover, DFO inhibited the enhanced therapeutic effect of RT with 5-ALA in hypoxia (Fig. S5) In contrast, IR with 5-ALA treatment impaired mitochondrial energy metabolism (Fig. 6e) and redox balance. Thus enhancement of RT effect by 5-ALA could be promoted by Fe^2+^ accumulation in mitochondria even in hypoxia.

In conclusion, our study revealed that intra-mitochondrial ROS production after RT was enhanced by 5-ALA-derived PpIX. 5-ALA-induced ROS burst provided a loss of MMP and mitochondrial dysfunction, causing mitochondria-dependent apoptosis. The mitochondrial damage inhibited cancer stemness in hypoxia, which might enables overcome hypoxia-induced RT resistance. These findings suggest that taking advantage of the radiosensitising effect of ALA-derived PpIX by combining 5-ALA with RT may be a novel strategy to improve the efficacy of RT for patients with PCA. Future clinical studies are considered to be important.

## Supplementary information

Reproducibility Checklist

Supplementary Figure. S1

Supplementary Figure. S2

Supplementary Figure. S3

Supplementary Figure. S4

Supplementary Figure. S5

Supplementary Table 1

## Data Availability

The data that support the findings of this study are available upon request from the corresponding author. The data are not publicly available due to privacy and ethical restrictions.
