# Supplementary figures and images for: 5-Aminolevulinic acid overcomes hypoxia-induced radiation resistance by enhancing mitochondrial reactive oxygen species production in prostate cancer cells
Source: Br J Cancer. 2022 Apr 1;127(2):350–63. doi: 10.1038/s41416-022-01789-4 (PMC9296661; doi:10.1038/s41416-022-01789-4)

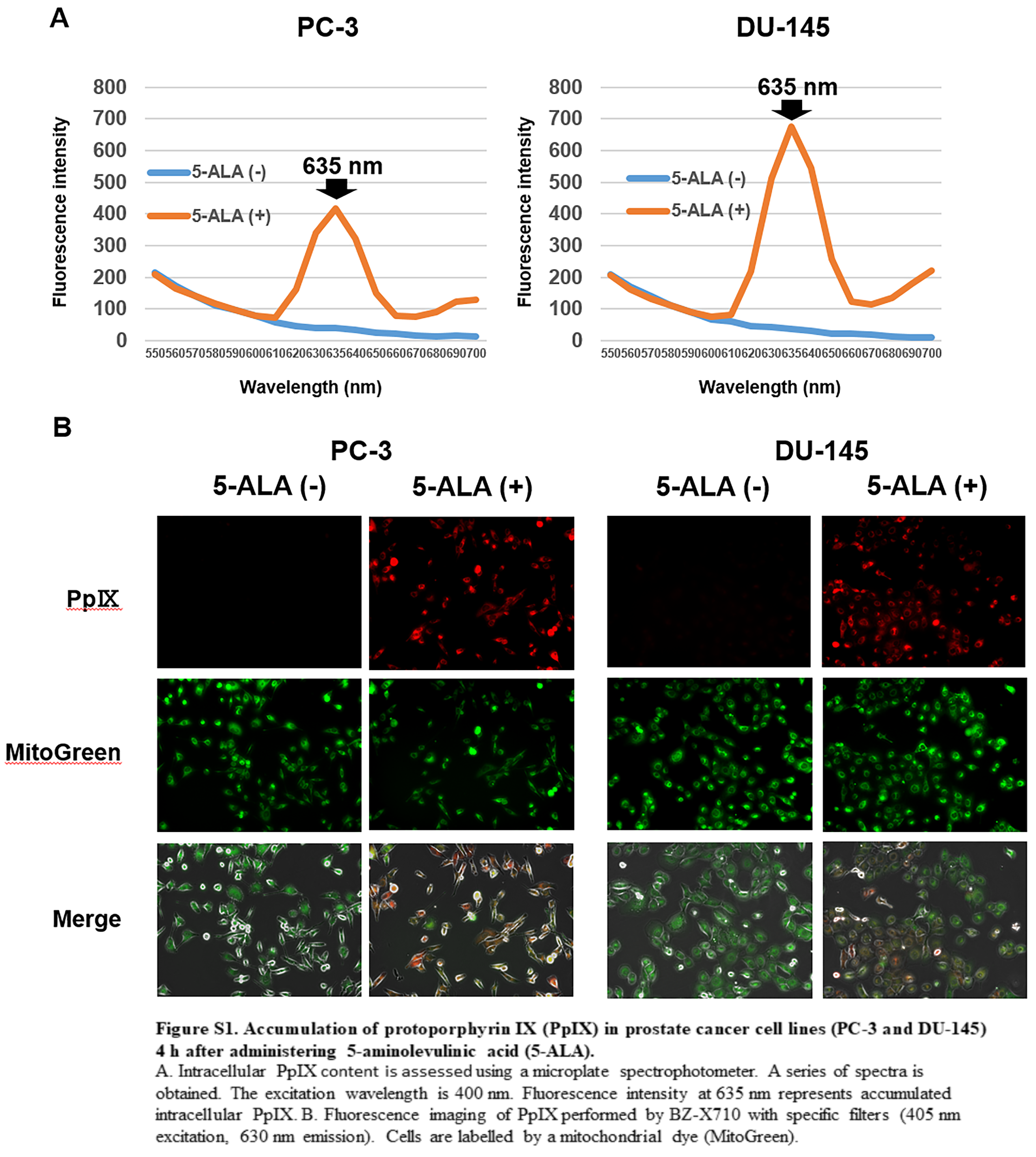

Supplement: Supplementary file 2 — Supplementary Figure. S1 [file 41416_2022_1789_MOESM2_ESM.tif]

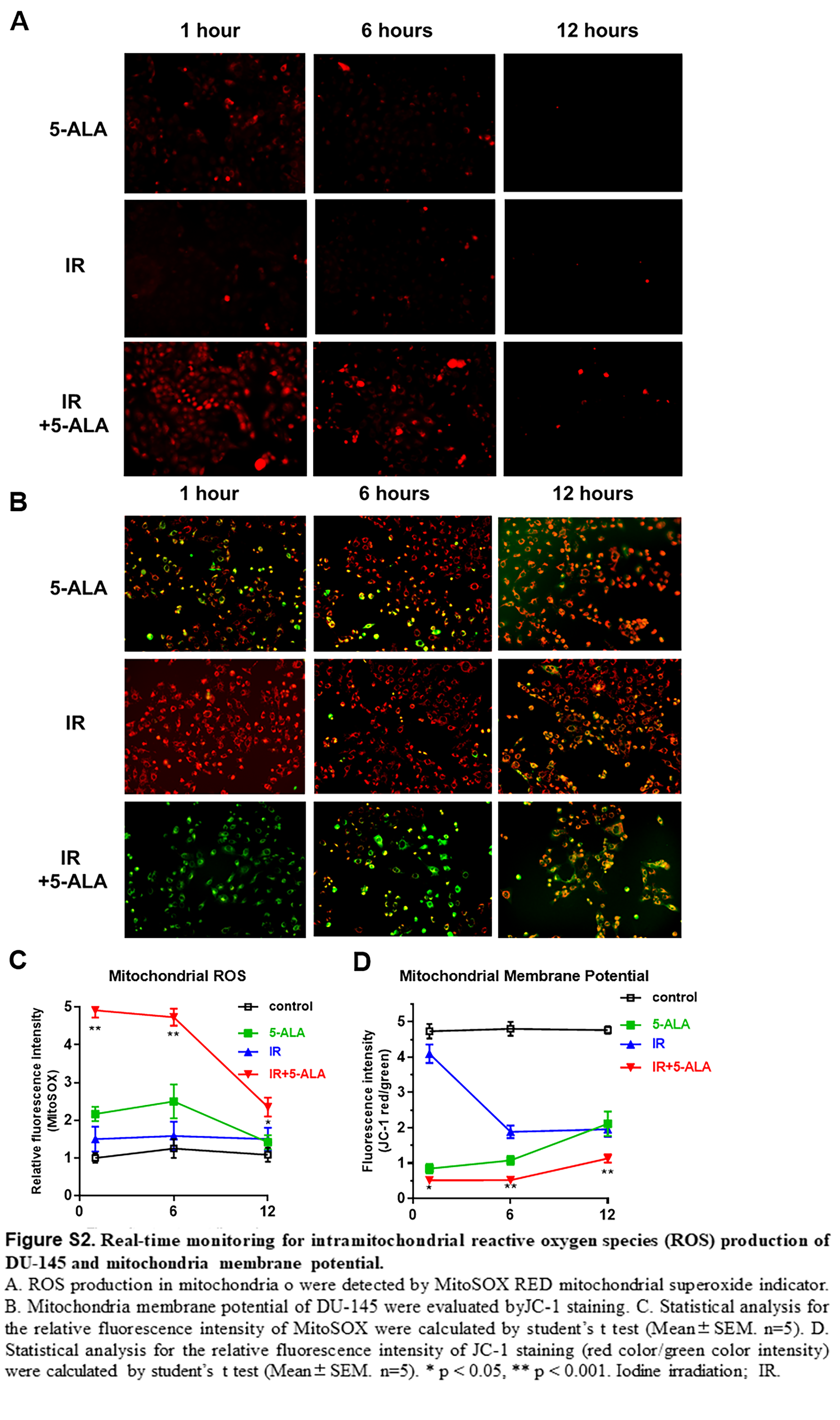

Supplement: Supplementary file 3 — Supplementary Figure. S2 [file 41416_2022_1789_MOESM3_ESM.tif]

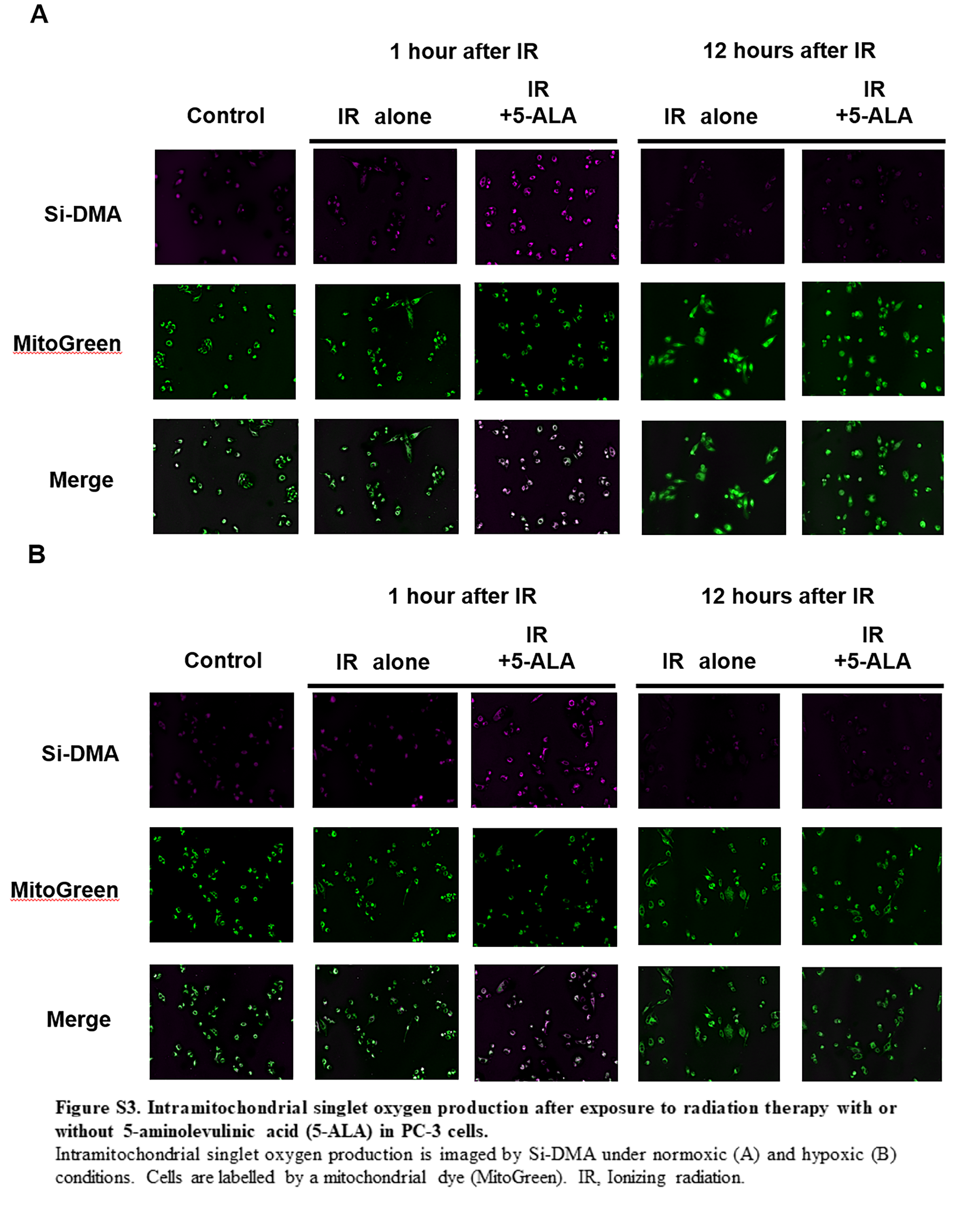

Supplement: Supplementary file 4 — Supplementary Figure. S3 [file 41416_2022_1789_MOESM4_ESM.tif]

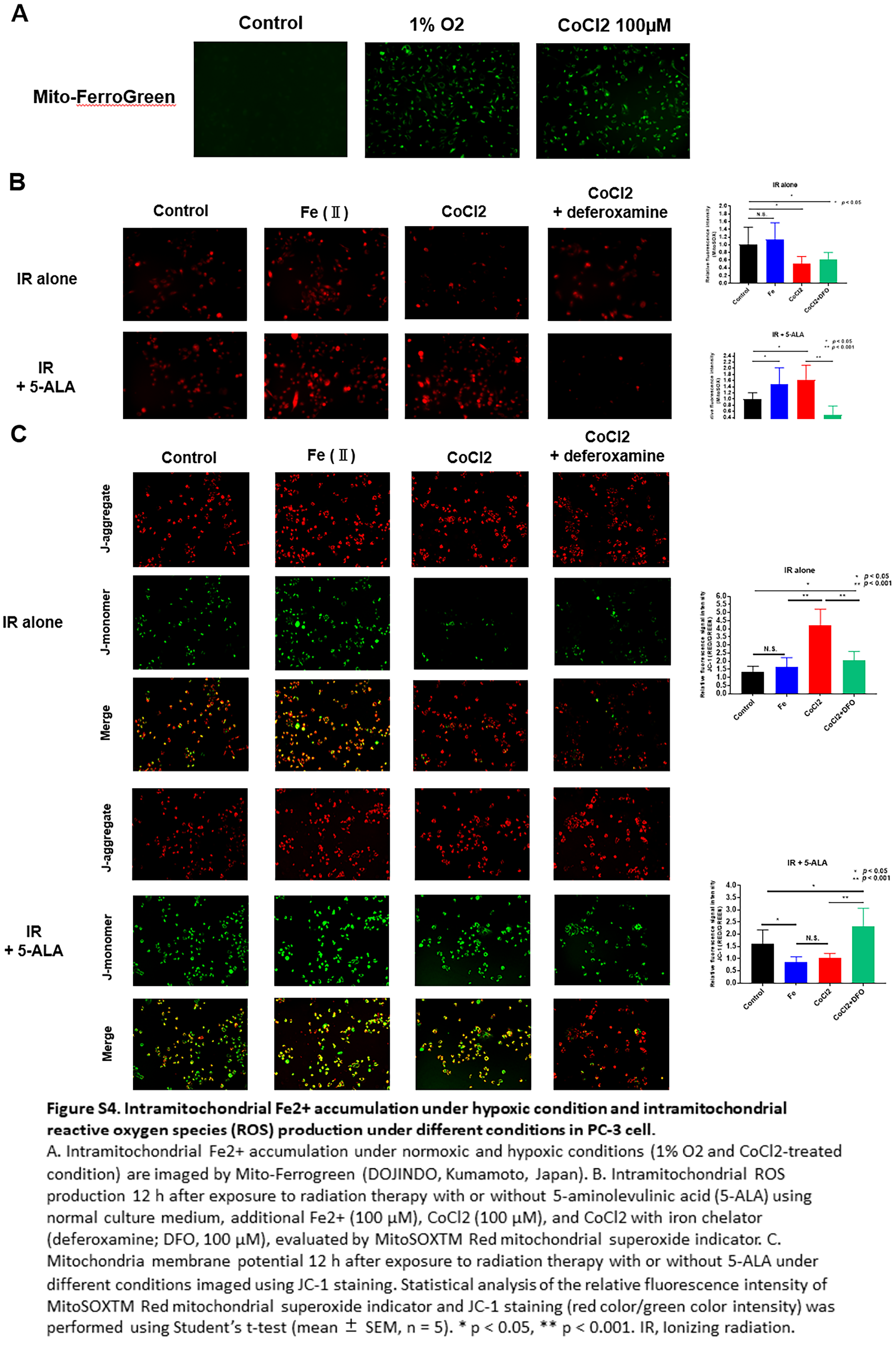

Supplement: Supplementary file 5 — Supplementary Figure. S4 [file 41416_2022_1789_MOESM5_ESM.tif]

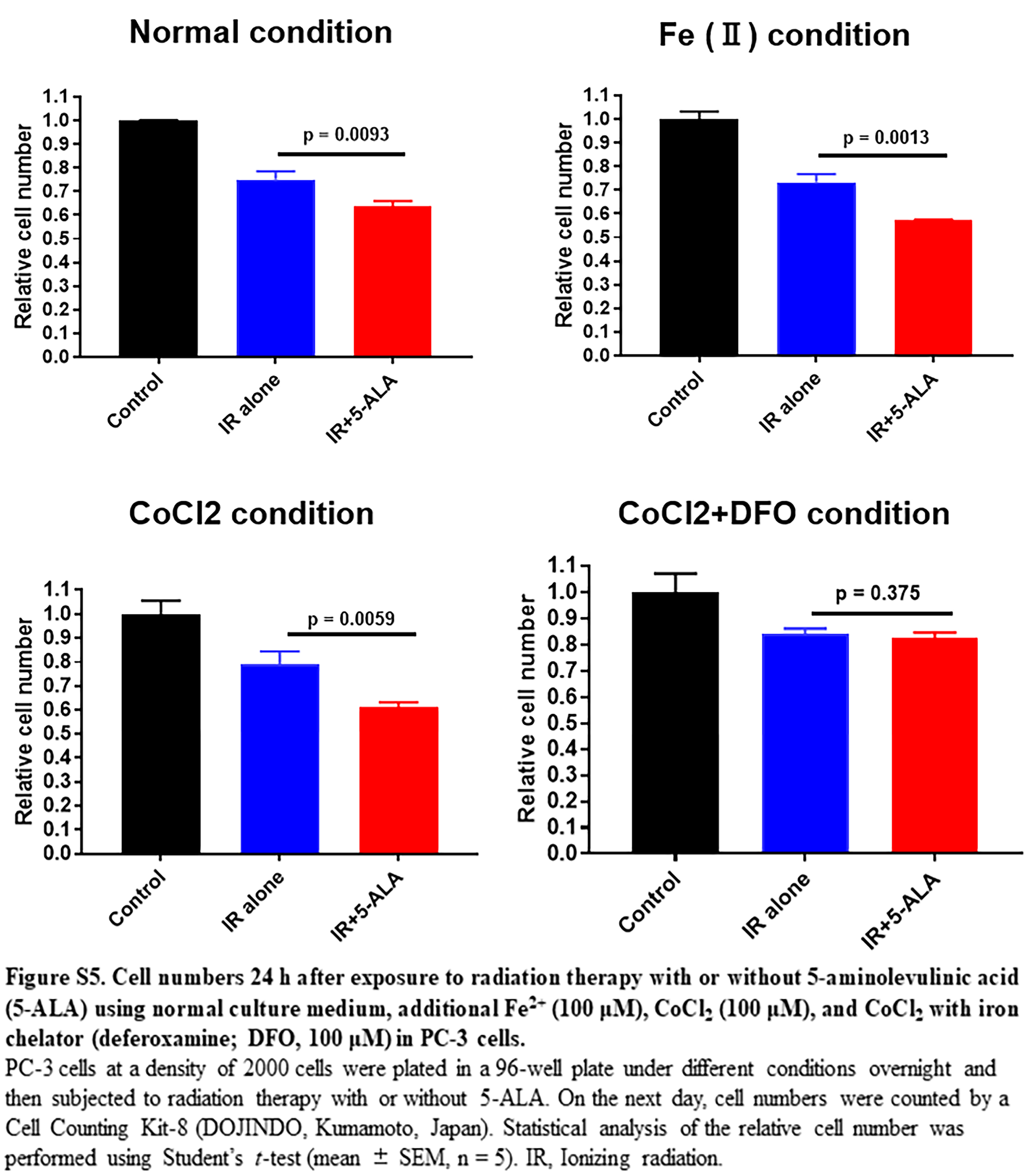

Supplement: Supplementary file 6 — Supplementary Figure. S5 [file 41416_2022_1789_MOESM6_ESM.tif]

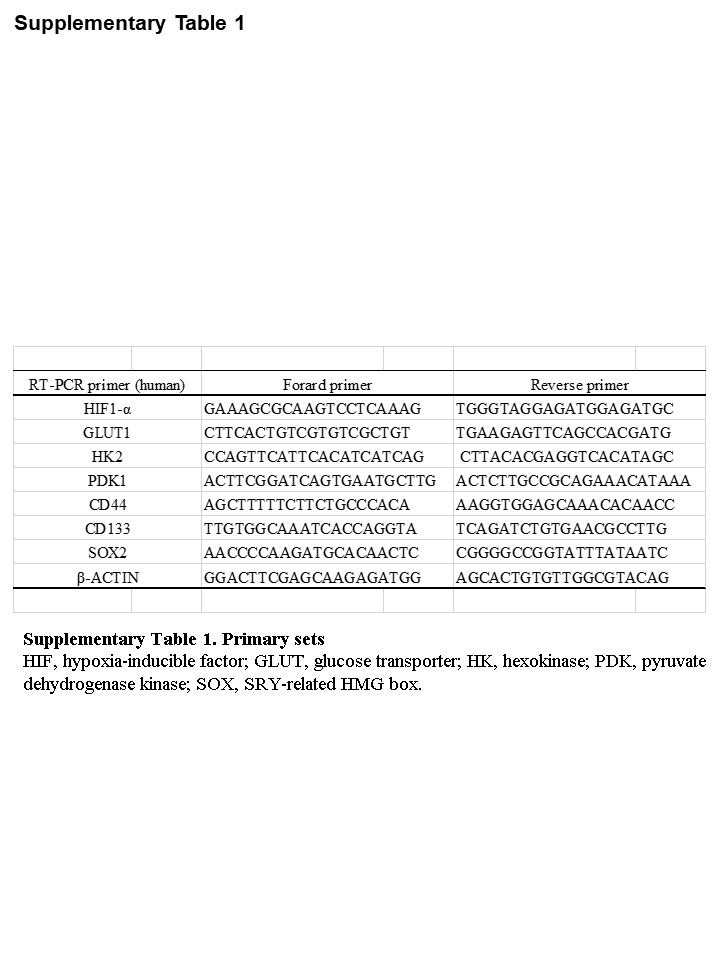

Supplement: Supplementary file 7 — Supplementary Table 1 [file 41416_2022_1789_MOESM7_ESM.tif]
